# Context-specific allocation of attentional control in anxiety and uncontrollability

**DOI:** 10.1007/s10339-026-01374-0

**Published:** 2026-07-11

**Authors:** Marcin Bukowski, Joaquín M. M. Vaquero, Alberto Acosta, Juan Lupiáñez

**Affiliations:** 1 Institute of Psychology, Jagiellonian University, Krakow, Poland; 2 Department of Experimental Psychology and Brain, Mind, and Behavior Research Center (CIMCYC), University of Granada, Granada, Spain

**Keywords:** Anxiety, Attentional control, Context-specific proportion congruency effect, Incidental learning, Uncontrollability

## Abstract

Volatile, unpredictable environments often evoke experiences of anxiety and uncontrollability. Previous research has shown that both high trait anxiety and a perceived lack of control can negatively affect the efficiency of attentional control. In the present research, we examined a novel hypothesis: that anxiety and uncontrollability impact the learning of control allocation in a context-dependent way. We assumed that highly anxious individuals or those who experience uncontrollability would exhibit reduced attentional control because incidental learning from contextual cues is altered. In Experiment 1, we investigated how dispositional differences in anxiety relate to incidental learning from the task context, indexed by the context-specific proportion congruency (CSPC) effect. Participants scoring high (vs. low) in trait anxiety did not differ in the magnitude of the CSPC effect during the initial learning phase. However, once the association was acquired, anxious participants showed difficulties in flexibly modifying it based on new contextual information indicating that the association between proportion congruency and the context was reversed. In Experiment 2, we examined the effect of induced uncontrollability on subsequent incidental learning from contextual cues. We observed an absence of the CSPC effect during the learning phase for participants who had previously been exposed to uncontrollability. We discuss the implications of these findings for theories concerning how anxiety and a lack of control influence flexible learning about when to allocate more or less attentional control based on contextual cues

## Introduction

When the environment changes unpredictably, previously learned and automatized knowledge may no longer be sufficient to ensure successful adaptation. In such cases voluntary and effortful control of actions must be engaged to flexibly adjust and modify existing behavioral patterns (Uddin 2021). However, as environmental demands for flexibility increase - requiring rapid and continuous adjustments to maintain fluent behavior - it can become difficult to cope with novelty and unpredictability in a conscious and effortful manner. It has been suggested that, in these situations, incidental (i.e., unintended) learning from contextual cues (Crump et al. 2006; Crump & Logan 2010; van Gaal et al. 2010) enables flexible adaptation without the need for effortful control. Thus, behavioral adjustment to incoming signals can rely on a form of contextual control that enhances flexibility while preserving smooth and effortless performance in changing environments. However, an important question remains: how does this process of incidental learning unfold when people’s basic needs for certainty and predictability are threatened?

Volatile and unpredictable environments can impose a sense of threat on individuals, often accompanied by feelings of anxiety or uncontrollability (Browning et al. 2015; Bukowski & Kofta 2017; Jonas et al. 2014). Environmental threats that evoke anxiety can impair executive control by inducing perceptual and behavioral competition, which often results in reduced performance (Pessoa 2009). Moreover, unsatisfied psychological needs can shape performance on tasks requiring cognitive control (Botvinick & Braver 2015). For example, a deprived sense of personal control over the environment has been shown to impair attentional control and cognitive flexibility (Bukowski et al. 2015; 2019; 2024). However, it remains unclear whether, and how, such threats influence incidental learning from contextual cues, and consequently the ability to flexibly adapt to environmental changes based on those cues. In the present work, we examine the impact of trait anxiety and experimentally induced uncontrollability on the context-specific allocation of attentional control. We propose that individuals who frequently experience anxiety or uncontrollability will exhibit lower levels of attentional control because their incidental learning from contextual cues (i.e., informative signals that help anticipate conflict trials) is disrupted.

### Anxiety and the allocation of attentional control

The relationship between anxiety and attentional functioning—particularly attentional control—has attracted interest from cognitive and clinical psychologists for decades (Beck & Clark 1997; Eysenck & Calvo 1992; Fox 1994; Shi et al. 2019). Early research proposed that anxiety constitutes a generalized state of emotional arousal that interferes with attention to environmental cues (Easterbrook 1959). Sarason (1975) argued that anxious individuals must divide their attention between internal, off-task cognitions (e.g., worry) and external task cues, in contrast to less anxious individuals who can focus more fully on task-relevant stimuli. More recent research has shown that anxiety is associated with reduced top-down control over threat-related distractors (Bishop et al. 2004). Similarly, Eysenck and others (2007) posited that anxiety impairs the efficient functioning of the goal-directed attentional system, thereby increasing the impact of the stimulus-driven system on information processing. Further research indicates that trait anxiety is linked to impoverished recruitment of prefrontal attentional control to inhibit distractors – even in the absence of threat-related stimuli (Bishop 2009). A comprehensive meta-analysis examining the relationship between anxiety and attentional control found that anxiety impairs attentional control efficiency (but not effectiveness), particularly in inhibition and switching, but not updating (Shi et al. 2019). Additionally, more recent research has provided converging evidence for Attention Control Theory, showing that higher levels of anxiety are associated with poorer performance on measures of set-shifting and inhibition (Messerlie et al., 2025).

A different line of research has examined anxiety beyond threat-related contexts, focusing on the functioning of the three attentional networks (orienting, alerting, and executive control). This work revealed that trait anxiety is associated with deficiencies in the executive control network, whereas state anxiety is linked to overactivation of the alerting and orienting networks (Pacheco-Unguetti et al. 2010). More recent findings show that differences in attentional network functioning between individuals high and low in trait anxiety are actually greater in the absence of threat-related information and diminish when such information is present (Pacheco-Unguetti et al. 2025). This pattern supports the conclusion that trait anxiety may be associated with heightened reactivity regardless of whether the environment is threatening or safe. Such over-reactivity to irrelevant environmental information may stem from difficulties in predicting when attentional control is needed to distinguish relevant from irrelevant information, and when it is not needed, given that some environmental interactions can be managed more automatically.

Browning and colleagues (2015) demonstrated that, compared with non-anxious individuals, those high in trait anxiety struggle to predict the occurrence of an electric shock in stable environments, performing no better than they do in volatile ones. Specifically, when learning action–outcome contingencies in either stable or volatile task environments, high-anxiety participants showed a reduced ability to adjust their outcome expectancies in response to changing conditions. In contrast, low-anxiety participants calibrated their predictions according to the volatility of the environment.

These results suggest that anxious individuals are more distracted by monitoring environmental changes – not only those related to threat (i.e., anxiety inducing stimuli). Such heightened monitoring may reflect an impairment in implicit learning based on contextual cues, resulting in an undifferentiated allocation of attentional control. Consistent with this account, a study on the attentional mechanisms of trait anxiety found that highly anxious individuals do not differentiate among contextual cues (i.e., safety, predictable threat, and unpredictable threat), instead allocating attention similarly across contexts (Stegmann et al. 2019). Building on this evidence, it can be expected that anxious individuals may also exhibit difficulties in incidental learning from contextual cues that guide the allocation of attentional control in neutral, non-threatening task environments.

### Uncontrollability and flexible learning from the environment

Unpredictable and uncertain environments diminish individuals’ sense of control over the outcomes of their actions (Bukowski & Kofta 2017; Kofta & Sedek 1999). Personal control can be defined as an organism's ability to produce desired outcomes through its actions and to avoid undesired ones (Skinner 1996). Uncontrollability occurs when there is objectively no contingency between one’s actions and the resulting outcomes (Seligman 1975). Prolonged and repeated experiences of uncontrollability can lead to learned helplessness, a state associated with negative cognitive, affective and motivational consequences (Hiroto & Seligman 1975). At the cognitive level, prolonged exposure to uncontrollability leads to difficulties in learning (e.g., avoiding harmful stimuli), making inferences, reasoning about relationships (i.e., updating information and integrating it into a coherent mental model), and employing more complex decision-making strategies (Kofta & Sedek 1998; Sedek & Kofta 1990; Sedek et al. 1993; von Hecker & Sedek 1999).

More recent studies showed that an induced state of uncontrollability can also lead to difficulties with attentional control (Bukowski et al. 2015) and cognitive flexibility (Bukowski et al. 2019; 2024). Specifically, Bukowski and colleagues (2015) showed that experimentally induced uncontrollability – produced through exposure to a series of unsolvable tasks – primarily impaired the efficiency of the executive control network, as evidenced by larger conflict effects. This impairment was accompanied by a less selective response strategy, particularly when no cues or spatially invalid cues were presented prior to the target stimuli, resulting in a reduced ability to disengage attention from invalid cues. Thus, induced uncontrollability was associated with less efficient functioning of the goal-driven attentional system (Bukowski et al. 2015). Other research showed that experienced uncontrollability can lead to higher task-switching costs and lower efficiency in various task-switching paradigms (Bukowski et al. 2019), as well as diminished flexible attentional shifting, measured by two different cognitive set-switching paradigms (Bukowski et al. 2024). Bukowski and Kofta (2017) proposed that the accumulation of behavioral uncertainty (i.e., uncertainty about how to act) underlies the detrimental effects of uncontrollability on attentional functioning. According to this account, repeated exposure to action–outcome noncontingency enhances the search for new information that may help resolve uncertainty about how to act efficiently. At the cognitive level, this can result in a broadened but dispersed focus of attention (i.e., reduced goal-directed attentional control), which further increases behavioral uncertainty and impedes effective action control.

This pattern of psychological functioning among individuals experiencing uncontrollability resembles that observed in highly anxious individuals, as both groups exhibit increased off-task cognitions (Mikulincer & Nizan 1988). Consistent with the cognitive interference hypothesis, exposure to unsolvable problems disrupts sustained selective attention and task performance, most likely due to heightened off-task thinking (Mikulincer 1989). If this is the case, then both anxious individuals and those exposed to prolonged uncontrollability should be less sensitive to incidental contextual information.

Recently, it has been argued that in highly uncontrollable and unpredictable environments, individuals may experience difficulties in forming effective action plans, resulting in a disorganized processing style characterized by a dysregulated balance between stability and distractibility (see Bukowski et al. 2024). Such dysregulation may impair performance on tasks that require both attention to contextual information and the application of top-down cognitive control based on that information. Prolonged exposure to unpredictability may further reduce the ability to learn from subtle contextual cues that signal how attentional control should be allocated. Consistent with this view, recent findings indicate that attentional dysregulation is a robust factor underlying a wide range of cognitive task impairments, including deficits in executive functioning, in adolescents with high anxiety (Roberts et al. 2023).

Although uncontrollability and anxiety are distinct psychological constructs, they appear to share a common feature: a maladaptive cognitive response to uncertain and unpredictable environments characterized by dysregulated attentional control. Accordingly, it is reasonable to expect that both highly anxious individuals and those repeatedly exposed to action–outcome non-contingency will exhibit difficulties in context-dependent learning about when to allocate more or less attentional control.

### Incidental learning from the environment: the case of context-specific congruency effects

Flexible behavior involves sensitivity to contextual cues that signal when behavioral adjustments are needed to better adapt to the environment. Learning about such cues can, in some cases, proceed incidentally (i.e., without intentional processing) and, in other cases, implicitly, as a by-product of experience or practice. This enables a smooth and flexible form of automatic performance guided by task context (Crump et al. 2008). Such automatic flexibility is exemplified by skilled musicians playing an instrument or athletes performing in dynamic sports environments.

The study of this type of automatic, flexible learning (D’Angelo et al. 2013) and the control of behavior can be addressed experimentally through the context-specific proportion congruency (CSPC) effect (Crump et al. 2006). In Stroop-like tasks, congruency effects refer to faster responses to congruent trials (e.g., the word *blue* printed in blue) than to incongruent trials. The magnitude of this effect is modulated by the overall proportion of congruent trials: the congruency effect is larger when most trials are congruent and reduced when most trials are incongruent. Importantly, these proportion-congruency effects can become associated with contextual cues (Crump et al. 2006, 2008).

Crump and colleagues demonstrated a context-specific proportion congruency (CSPC) effect in which high and low proportions of congruent trials were associated with different target locations (Crump et al. 2006). For example, most trials may be incongruent when the target appears in the upper location of the screen, whereas most trials are congruent in the lower location. Participants learn this association between location and proportion congruency, as reflected in a smaller congruency effect for targets presented in the upper compared to the lower location (Crump et al. 2006). This reduction is interpreted as evidence that participants allocate greater attentional control in contexts with a higher proportion of incongruent trials. Accordingly, a reduced congruency effect at a given location is taken as evidence of learning the context–proportion congruency association and adjusting attentional control based on this learning.

Subsequent research has shown that social categories (e.g., female vs. male faces) can also be associated with different proportions of congruent trials, thereby serving as social contexts for the allocation of attentional control (Cañadas et al. 2013). Notably, participants appear to implicitly learn to allocate greater attentional control to contexts associated with higher conflict (i.e., where incongruent trials are more frequent), resulting in reduced interference in those contexts.

### Current research

Trait anxiety and perceived uncontrollability can both arise from exposure to unpredictable environments and from ineffective coping attempts. Previous research has shown that individuals high in anxiety, as well as those who experience a lack of control, exhibit less efficient goal-directed attentional control (see Bukowski et al. 2015; Pacheco-Unguetti et al. 2012; 2025).

In this research, we investigate how anxiety and uncontrollability may influence incidental learning processes involved in the allocation of attentional control. Previous studies have shown that, although such learning processes are largely involuntary, they can be modulated by motivational factors, such as achievement motivation or the motivation to avoid loss, in tasks like artificial grammar learning or implicit sequence learning (Chon et al. 2018; Eitam et al. 2008). Based on this evidence, we hypothesized that anxiety and uncontrollability - conceptualized as aversive experiences arising from threats to fundamental motives of predictability and control (Bukowski & Kofta 2017; Jonas et al. 2014) – could similarly alter performance on tasks that rely on incidental learning from contextual cues.

How can anxiety or uncontrollability affect flexible, incidental learning from the context? We addressed this question by selecting participants scoring high vs. low in anxiety trait (Experiment 1) or participants exposed to uncontrollable (vs. controllable) task environments (Experiment 2). Subsequently we measured their ability to learn from contextual cues regarding where to allocate their attentional control. Specifically, we hypothesized that chronic anxiety would be associated with a decreased reliance on contextual cues, resulting in a reduced CSPC effect among highly anxious participants. On the other hand, experienced uncontrollability is thought to impair efficient attentional selection, likely due to elevated levels of behavioral uncertainty (Bukowski et al. 2015; Bukowski & Kofta 2017). This impairment could manifest as a decreased tendency to learn from environmental cues about where to allocate attentional control, resulting in a reduction of the CSPC effect.

Thus, both dispositional anxiety and situational uncontrollability may be associated with less efficient incidental learning from context. Based on these considerations, we propose that anxious individuals and those experiencing uncontrollability might show similar difficulties in predicting the probable outcomes of their actions and in learning from a constantly changing environment. These difficulties may stem from prior learning that the environment does not provide valid cues to guide efficient action and goal pursuit. Consequently, anxiety and uncontrollability could impair the selective attentional processes required for incidental learning from contextual cues. Accordingly, we expect to observe diminished CSPC effects – reflecting reduced sensitivity to environmental changes – and impaired contextual learning in both highly anxious participants and those exposed to uncontrollability.

## Experiment 1

In this study, we examined how dispositional differences in anxiety relate to incidental learning regarding when to allocate more or less attentional control. We focused on participants differing in trait anxiety and assessed their performance on a modified flanker task, in which the location of the arrows served as a cue for the proportion of congruent trials. This design enabled us to test for differences in the CSPC effect. Furthermore, because the task included both a learning phase and a re-learning phase—during which the cue-location associations were reversed—we were able to investigate the role of flexible learning in forming new associations.

## Method

### Participants

For participant’s recruitment we followed the same procedure as used in our previous studies (Pacheco-Unguetti et al. 2010; Pérez-Dueñas et al. 2014). Fifty-four university students (forty-two females) aged 18 to 32 years (*M* = 22.13; *SD* = 3.3), participated in this study. They were recruited for exhibiting high or low scores on the State-Trait Anxiety Inventory (STAI-T; Spielberger et al. 1982) from a large sample of students. Participants completed this questionnaire, along with other affective measures, between four and ten months before the lab session. The High Anxiety trait group (HA) was composed of 24 participants (18 females) and the Low Anxiety trait group (LA) by 30 participants (24 females). The STAI-T was administered again in the lab session (then the Mean and SD values were 30.4 (5.9) for HA and 17.5 (7.2) for LH). A score of 30 corresponds to the 70th percentile for women and the 85th percentile for men in the Spanish population, whereas a score of 17 corresponds to the 25th percentile for women and the 40th percentile for men.

A priori statistical power analyses were not performed for this study. Therefore, we conducted a sensitivity analysis using G*power (Faul et al. 2007), which focused on the difference between two independent means (between the High vs. Low Anxiety groups) for the main CSPC index. Results showed that for the final sample size of N = 54 the minimum effect size that could be detected for α = 0.05 and β = 0.80 is *d* = 0.69.^1^

The experiment was part of a larger research project approved by the University of Granada Ethical Committee (356/CEIH/2018) and adhered to the ethical standards of the Declaration of Helsinki. Informed consent was obtained from the participants, who were either paid 8€ or participated in exchange of course credits.

### Materials and apparatus

We used the Spanish version of the STAI (Spielberger et al. 1982). This questionnaire assesses both state and trait anxiety, including 20 items on a 0–3 rating scale for each form. State anxiety items ask how anxious “you feel right now”, with scale values representing: *Nothing* (0), *Some* (1), *Quite* (2), *Much* (3). Trait Anxiety items address “how anxious do you feel in general”, with the rating scale comprising: Rarely (0), Sometimes (1), Often (2), Almost Always (3).

In the lab session participants performed a flanker task, which was run on E-Prime 2.0 software and presented on a 17-inch computer screen. The stimuli for this task consisted of five black arrows arranged horizontally on a grey background. These arrows were centered along the horizontal axis and could appear either above or below a fixation point placed at the center of the screen.

### Procedure

In the lab session, participants firstly filled the STAI-S form, then carried out the flanker task (Eriksen & Eriksen 1974), and finally completed the STAI-T form. As mentioned, in each trial of the flanker task five black arrows appeared either above or below the fixation point. Participants were asked to respond whether the central arrow (target) was oriented towards the right or left side. The four flanker arrows (non-target stimuli) always pointed in the same direction; thus, trials were congruent if the central arrow pointed in the same direction as the flankers, or incongruent if the central arrow pointed in the opposite direction. Participants were instructed to ignore the flankers to focus on the central (target) arrow. Each trial began with a centrally located fixation cross displayed for a variable time between 400 and 1600 ms. Then, the five arrows were presented either above or below the fixation cross and remained in the screen until a response was made or for a maximum of 3000 ms. Finally, the fixation cross reappeared for enough time to complete a total of 4600 ms for each trial. Participants used a QWERTY keyboard, pressing the “Z” key with the index finger of their left hand for left-side responses and the “M” key with the index finger of their right hand for right-side responses.

To ensure an experimental procedure suitable for measuring the CSPC effect, we manipulated the association between the location of the arrows (above or below the fixation point) and the proportion of congruent versus incongruent targets. The target location on each trial was randomized, with overall congruence at 50% for both congruent and incongruent trials. However, one location was associated with a high proportion of congruence (80% congruent trials), while the other was associated with a low proportion (20% congruent trials). The session consisted of two parts: in the first half, one location (above or below the fixation) was associated with a high proportion of congruence, while the other was associated with low congruence. In the second half, this association was reversed. The assignment of which location was first associated with high or low congruence was counterbalanced across participants.

Participants completed 40 practice trials and then 10 blocks of 40 experimental trials each one. In the first five blocks, the first location-proportion congruence association was introduced, which was considered the Learning Phase. The second five blocks, in which the opposite association was implemented, were termed the Re-Learning Phase, as participants had to learn that proportions of congruency conditions associated with each spatial location had been reversed.

## Results

### Anxiety measure

As shown in Table 1, at the time of the experimental session, STAI-T scores were higher for the HA group (*M* = 30.38, *SD* = 5.74) than the LA group (*M* = 17.53, *SD* = 7.26), *t*(52) = 6.94, *p* < 0.001, *d* = 1.90, while there were no statistically significant differences in STAI-S scores between the two groups, *t*(52) = 1.45, *p* = 0.15, *d* = 0.40 (HA: *M* = 18.21, *SD* = 10.72; LA: *M* = 14.57, *SD* = 7.66).

**Table 1.** Means and Standard Deviations for Age, STAI-S, and STAI-R by Group and Gender

		N	Age	State Anxiety	Trait Anxiety
HA		24	22.21 (3.3)	18.21 (10.72)	30.38 (5.86)
Female		18	22.34 (3.07)	20.11 (11.36)	31.33 (5.08)
Male		6	21.84 (3.89)	12.50 (6.16)	27.50 (7.56)
LA		30	22.01 (3.29)	14.57 (7.66)	17.53 (7.38)
Female		24	21.83 (3.34)	15.67 (7.86)	18.54 (7.28)
Male		6	23 (2.94)	10.17 (5.27)	13.50 (6.92)

*HA* High Anxiety, *LA* Low Anxiety

### Reaction time analyses

As usual in similar flanker tasks (Luna et al. 2021), the RT analyses excluded trials with incorrect responses and outliers (2.44%), defined as RTs under 200 ms or above 1500 ms (0.51%). RTs for the ten experimental trials blocks were analyzed using a mixed ANOVA with Congruency (congruent vs. incongruent), Proportion Congruent (High Proportion Location vs. Low Proportion Location) and Phase (learning vs. re-learning) as within-participants factors, and the group variable based on the anxiety trait scores (HA vs. LA). The main effect of Congruency was significant,* F*(1,52) = 365.47, *p* < 0.001, *η*^*2*^_*p*_ = 0.875, showing that responses for congruent trials (550 ms) were faster than for incongruent trials (642 ms). Importantly, the Proportion Congruent by Congruency interaction was significant *F*(1, 52) = 17.79, *p* < 0.001, *η*^*2*^_*p*_ = 0.255, indicating that the congruency effect for the High Proportion Location (HPL = 102 ms) was higher than for the Low Proportion Location (LPL = 83 ms). Therefore, there was evidence of a CSPC effect (see Table 2).

**Table 2:** Reaction Times and Accuracy as a Function of Group, Learning Phase, Trial Type, and Proportion Congruency

		Learning Phase						Re-Learning Phase					
		HPC		LPC		CSPC		HPC		LPC		CSPC	
		RT	ER	RT	ER	RT	ER	RT	ER	RT	ER	RT	ER
HA	CON	558	0.5	566	0.6			559	0.5	565	0.4		
	INC	670	6.5	650	3.3			645	4.8	655	5.2		
	CE	112	6.0	84	2.7	28	3.3	86	4.3	90	4.8	-4	-0.5
LA	CON	535	0.4	537	0			535	0.3	553	0.5		
	INC	637	6.3	617	4.1			643	5	631	4.1		
	CE	102	5.9	80	4.1	22	1.8	108	4.7	78	3.6	30	1.1

*HA* High Anxiety, *LA* Low Anxiety, *CON* Congruent trial, *INC* incongruent trial, *ACC* Accuracy (error rates), *HPC* High proportion congruent, *LPC* Low proportion congruent, *CE* Congruency Effect. *CSPC* Context-Specific Proportion Congruency effect. Reaction times (RTs) and error rates (ERs) are presented for each group (high vs. low anxiety) as a function of learning phase (learning vs. re-learning), trial type (congruent vs. incongruent), and proportion congruent location (high vs. low proportion congruent). Congruency effects (CE; congruent – incongruent trials) and context-specific proportion congruency effects (CSPC; CE in high-proportion congruent minus CE in low-proportion congruent locations) are also reported

**Fig. 1:**
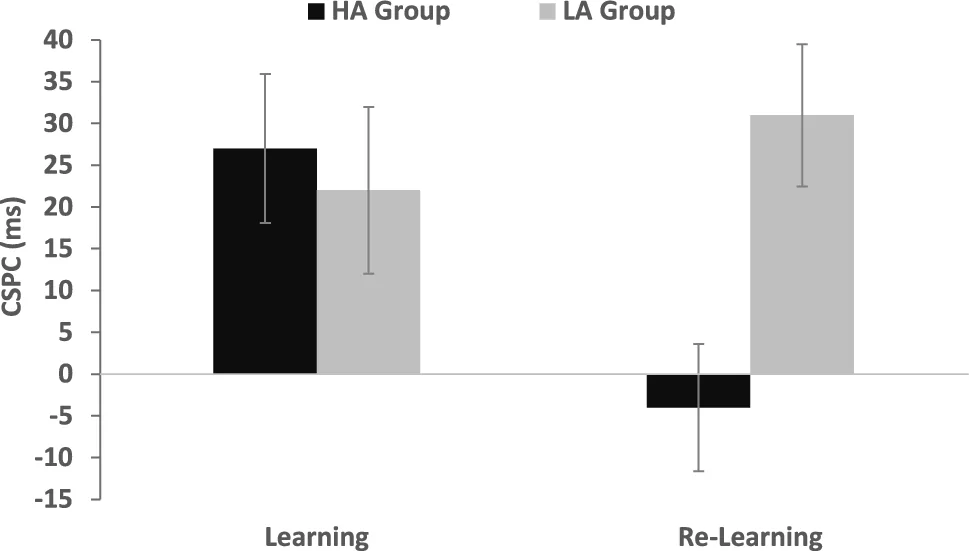
Context-Specific Proportion Congruency Effect as a Function of Trait Anxiety and Learning Phase. Note. *CSPC* context-specific proportion congruency; *HA* high anxiety; *LA* low anxiety. Error bars represent standard error of the mean (SEM)

Neither the Group x Congruency interaction (*F* < 1) nor the second order interaction Group x Proportion Congruent x Congruency (*F*(1, 52) = 2.57, *p* = 0.115, *η*^*2*^_*p*_ = 0.047) were significant. However, the four-way interaction of Group x Proportion Congruent x Congruency x Phase reached significance, *F*(1, 52) = 5.54, *p* = 0.022, *η*^*2*^_*p*_ = 0.096. As shown in Fig. 1, the CSPC effect (i.e., the difference in the Congruency effect between the two locations), was present for both groups in the Learning Phase, but it was only exhibited by the LA group in the Re-Learning Phase. Accordingly, an ANOVA performed on each Phase revealed that the Proportion Congruent x Congruency was significant in both the Learning, *F*(1, 52) = 14.13, *p* < 0.001, *η*^*2*^_*p*_ = 0.214, and the Re-Learning Phase, *F*(1, 52) = 5.29, *p* = 0.025, *η*^*2*^_*p*_ = 0.092. However, the three-way Group x Proportion Congruent x Congruency interaction was significant in the Re-Learning Phase, *F*(1, 52) = 9.18, *p* = 0.004, *η*^*2*^_*p*_ = 0.150, but not in the Learning Phase (*F* < 1). In fact, in the Learning Phase the Proportion Congruent X Congruency interaction (CSPC effect) was significant, and quite similar, in both groups (28 ms for HA group, *F*(1, 23) = 9.62, p = 0.005, *η*^*2*^_*p*_ = 0.299; 22 ms for LA group, *F*(1, 29) = 5.50, *p* = 0.026, *η*^*2*^_*p*_ = 0.160). In contrast, in the subsequent Re-Learning Phase the Proportion Congruent x Congruency interaction was significant only for the LA group (30 ms, *F*(1, 29) = 19.91, *p* < 0.001, *η*^*2*^_*p*_ = 0.407) and absent in the HA group (-4 ms, *F* < 1). All the remaining non-significant effects were *p* > 0.15, except the marginally significant interaction Group x Phase, *F*(1, 52) = 3,56, *p* = 0.06, *η*^*2*^_*p*_ = 0.064. However, further analyses of this interaction did not reveal any other significant effect (all *p* > 0.08).

The CSPC effect was directly analyzed by measuring the RT differences in the Congruency effect between high and low proportion congruency contexts (locations). A mixed ANOVA performed on this index, including the factors Learning Phase and Group, confirmed a significant interaction effect, *F*(1, 52) = 5.54, *p* = 0.022, *η*^*2*^_*p*_ = 0.096. Specifically, the group factor failed to reach significance in the Learning Phase, *F*(1, 52) = 0.17, *p* = 0.685, *η*^*2*^_*p*_ = 0.003, but it was significant in the Re-Learning Phase, *F*(1, 52) = 9.18, *p* = 0.004, *η*^*2*^_*p*_ = 0.150.

A one-tailed Student’s t-test comparing the CSPC index of HA and LA groups in the Learning Phase was not significant, *t*(52) = 0.41, *p* = 0.685, *d* = 0.11. However, in the Re-Learning phase the t-test revealed a significant effect, *t*(52) = - 3.03, *p* = 0.004, *d* = - 0.83, which is larger than the *d* = 0.29, indicated as the minimum effect size that could be detected with our sample according to the above reported sensitivity analysis.

The same conclusions can be stated by means of Bayesian Statistic analyses, showing evidence in favor of the non-difference hypothesis in the Learning Phase, BF10 = 0.295, and support for a between groups difference in the Re-Learning Phase, BF10 = 10.348.

### Accuracy analyses

We conducted a mixed repeated measures ANOVA also on errors rates. The analysis revealed significant effects of Congruency, *F*(1, 52) = 42.257, MSE = 0.216, *p* < 0,001, *η*^*2*^_*p*_ = 0.448, and Proportion Congruency, *F*(1, 52) = 8.80, *p* = 0.005, *η*^*2*^_*p*_ = 0.145, as well as the interaction between these two factors, *F*(1, 52) = 7.45, *p* = 0.009, *η*^*2*^_*p*_ = 0.125. The second order interaction Proportion Congruent x Congruency x Phase did not reach significance (*F* = 1.33) and neither did all the interactions involving the Group factor (all *F* < 1). Nevertheless, as shown in Table 2, the error rates indicated a similar pattern to the RTs. In particular, the congruency effects observed for error rates were numerically higher in HPC than LPC for both groups in the Learning Phase but only for the LA group in the Re-Learning Phase. Therefore, there was no evidence of trade-off effects between RT and accuracy measures.

A one-tailed Student’s t-test comparing the CSPC index of HA and LA groups in the was not significant neither for the Learning Phase, *t*(52) = 0.68, *p* = 0.502, *d* = 0.19, nor for the Re-Learning phase, *t*(52) = - 0.69, *p* = 0.491, *d* = - 0.19.

The same conclusions can be stated by means of Bayesian Statistic analyses, showing evidence in favor of the non-difference hypothesis in the Learning Phase, BF10 = 0.333 and in the Re-Learning Phase, BF10 = 0.336.

### Inverse efficiency in the flanker task

Given the apparently similar pattern of results observed in RT and accuracy data, a combined analysis was performed on the Inverse Efficiency Score (Bruyer & Brysbaert 2011; IES = Mean RT / % correct) as a dependent variable. The four-way interaction Group x Learning Phase x Proportion Congruent x Congruency failed to be significant, *F*(1, 52) = 2.76, *p* = 0.10, *η*^*2*^_*p*_ = 0.050. An ANOVA performed on each phase replicated the results observed for RT. Whereas in the Learning phase only the Congruency x Proportion Congruent interaction was significant, *F*(1, 52) = 10,89, *p* = 0.002, *η*^*2*^_*p*_ = 0.173, which was not modulated by Group (*F* < 1)**,** the Re-Learning phase showed a significant Congruency x Proportion Congruent x Group interaction, *F*(1, 52) = 4.72, *p* = 0.035, *η*^*2*^_*p*_ = 0.08. In this Re-Learning phase, only the LA group showed a CSPC effect, *F*(1, 29) = 7,722, *p* = 0.009, *η*^*2*^_*p*_ = 0.210 (*F* < 1 for the HA group).

A one-tailed Student’s t-test comparing the CSPC index of HA and LA groups in the Learning Phase was not significant, *t*(52) = 0.91, *p* = 0.365, *d* = 0.25. However, in the Re-Learning phase the t-test revealed a significant effect, *t*(52) = - 2.17, *p* = 0.034, *d* = - 0.60.

Similar conclusions can be drawn from the Bayesian analyses, showing evidence in favor of the non-difference hypothesis in the Learning Phase, BF10 = 0.389, and weak support for a between groups difference in the Re-Learning Phase, BF10 = 1.859.

## Discussion

In summary, we expected to observe reduced CSPC effects in participants with high trait anxiety. However, group differences in the CSPC effect emerged only during the re-learning phase but not in the learning phase. This finding indicates that highly anxious participants could learn associations based on contextual cues but were less able than those with lower anxiety to adjust their responses when the task context changed. These results seem to suggest that the tendency of individuals high in trait anxiety to apply attentional control does not impede initial learning and may even facilitate rapid acquisition. However, once an association is established, anxious participants demonstrate reduced flexibility in modifying it according to contextual information. Thus, our findings provide initial evidence for a diminished ability to re-learn – but not to learn initially – from the immediate context among highly anxious participants. However, it is important to acknowledge that the effect for the re-learning phase was not anticipated and therefore it has an exploratory status.

Although not predicted, this interesting result can be meaningfully interpreted considering previous research on anxiety and attentional control. The attentional functioning of anxious participants is more stimulus-driven, characterized by a higher activation of the alerting and orienting networks (Pacheco-Unguetti et al. 2010). This may explain why anxious participants remain sensitive to incidental environmental cues and rely on automatic processing. Paradoxically, this weaker goal-directed control may have prevented a decrement in the CSPC effect during the initial learning phase, as attention was more externally oriented. However, when controlled processing was required, as in the re-learning phase, performance declined.

This pattern of results can also be explained with reference to the revised reinforcement sensitivity theory (Gray & McNaughton 2000), which posits that trait anxiety is primarily associated with heightened sensitivity of the Behavioral Inhibition System (BIS), leading to enhanced conflict detection and resolution. Highly anxious individuals, characterized by increased conflict monitoring and vigilance, may be more likely to detect contextual cues during the learning phase. However, sustained vigilance combined with reduced inhibitory control may impede the successful acquisition of new associations.

Consistent with this account, research in clinical psychology indicates that individuals high in trait anxiety or intolerance of uncertainty exhibit reduced flexibility in learning, particularly in updating threat–safety associations. This is reflected in impaired discrimination between threat and safety cues and persistent responding to previously threatening stimuli during extinction (Morriss et al. 2021). More recent findings further suggest that highly anxious individuals experience difficulties in overcoming previously learned responses (i.e., resolving interference from prepotent responses in neutral contexts; see Wilson et al. 2018).

Taken together, these findings suggest that reduced cognitive flexibility – and, relatedly, difficulties in updating previously learned associations – may have impaired performance during the re-learning phase of the Flanker task, rather than reflecting a general deficit in attentional control.

## Experiment 2

In this next experiment, we studied the effect of uncontrollability experiences on subsequent incidental learning from the task environment. Previous research showed that experimentally induced uncontrollability affects goal-driven attentional control (Bukowski et al. 2015). In this experiment, we focused on incidental, yet flexible learning based on implicit contextual cues, which does not rely on explicit goals to guide performance. Specifically, we investigated how uncontrollability - induced through a paradigm of action–outcome non-contingency learning - affects the CSPC effect, using the same procedure as in Experiment 1. We primarily hypothesized that high levels of uncontrollability would reduce the ability to learn from contextual cues. Additionally, we examined both learning and re-learning phases, as previous research suggests that a lack of personal control can impair the cognitive flexibility required to adjust information-processing strategies in response to changing task contexts (Bukowski et al. 2019, 2024). Additionally, we focused on the performance in the re-learning phase as it can allow us to compare the effects of uncontrollability with the findings obtained in Experiment 1 when individual differences in anxiety were assessed.

## Method

### Participants

Eighty-two university students (fifty-nine females) aged from 18 to 32 years (*M* = 20.96, *SD* = 2.45) participated in this experiment and were randomly assigned to the uncontrollability (*N* = 39) or controllability (*N* = 43) induction conditions of the *Informational Helplessness Training (IHT;* Sedek & Kofta 1990). Following previous research (Bukowski et al. 2015), we applied exclusion criteria based on participants’ responses to manipulation check items assessing perceptions of control, efficacy, and task difficulty related to performance on the IHT task. Specifically, four participants in the uncontrollable group whose reported levels of efficacy or control were above the midpoint of the scale (3), and three participants in the controllable group who reported levels of efficacy or control below the midpoint, were excluded from further analyses. Additionally, one participant from the experimental group was excluded for committing more than 30% errors on the Flanker task. Consequently, the final sample consisted of 74 participants: 34 in the experimental (uncontrollability) group and 40 in the control (controllability) group.

A priori statistical power analyses were not performed for this study. Therefore, we conducted a sensitivity analysis using G*Power (Faul et al. 2007), which focused on the difference between two independent means (between experimental groups) for the main CSPC index. Results showed that for the final sample size of N = 74 the minimum effect size that could be detected for α = 0.05 and β = 0.80 is *d* = 0.59.^2^

The experiment was part of a larger research project approved by the University of Granada Ethical Committee (356/CEIH/2018) and adhered to the ethical standards of the Declaration of Helsinki. Informed consent was obtained from the participants, who took part in this experiment in exchange of course credits.

### Materials and apparatus

Participants performed a computerized version of the IHT control manipulation, and the flanker task previously used in Experiment 1. Both tasks were conducted using E-Prime 2.0 software and presented on a 17-inch computer screen. After the IHT procedure, participants were asked to answer five manipulation check items aimed at determining whether the uncontrollability and controllability conditions were properly induced. These items used a 7-point Likert-type scale and assessed the current affective state (ranging from negative to positive), arousal, perceived difficulty, self-efficacy (to what extent they think that the task has been solved correctly), and perceived control during the IHT task (with answers ranging from 1—absolutely no control, to 7—full control).

### Procedure

In the lab session participants firstly performed the Informational Helplessness Training (IHT), then completed the five manipulation check items, and finally carried out the same flanker task from Experiment 1. The IHT is an experimental task designed to induce experiences of uncontrollability and controllability. We applied a computerized version of the IHT procedure, which was previously successfully used to induce experiences of uncontrollability and controllability but also to impact cognitive performance on subsequent tasks (Bukowski et al. 2015, 2024; Kofta & Sedek 1999; Sedek & Kofta 1990). The IHT procedure consisted of a series of six discrimination problems which were presented one after another. Each problem consisted of 12 trials, with one figure displayed on the screen for each trial. Across all trials, the figures varied on five dimensions: 1) size (small or large), 2) shape (triangle or circle), 3) surface (plain or striped), 4) position of a line (at the top or bottom of the figure), and 5) size of the letter ‘r’ in the middle of the figure (small or large). The participants’ task was to identify the diagnostic feature of the figures in each discrimination problem (e.g. a small letter ‘r’). The task was presented as solvable to all participants. They were told that by using a cue (‘yes’ or ‘no’ below the figure presented on the screen), they could identify the feature of the figure that constitutes the solution of the problem. The instructions explained that ‘yes’ indicates presence (e.g., the presented circle with the small “r” letter has the diagnostic feature), whereas ‘no’ indicates absence of the target feature in the figure (e.g., the triangle with the large “R” letter does not have the diagnostic feature).

After completing each problem, a list of ten features (i.e., possible solutions) was presented to participants, and they were asked to indicate the solution by pressing the corresponding key on the computer keyboard. In the uncontrollability condition, the sequence of 'yes' and 'no' cues was arranged so that each possible hypothesis for a problem solution received 50% confirmatory and 50% non-confirmatory evidence, making it impossible to solve any of the six tasks. In contrast, in the controllability condition, participants received information that allowed them to solve all six tasks accurately. In neither condition were participants informed about their success or failure after completing consecutive problems.

## Results

### Manipulation checks

As expected, participants in the control group perceived higher control and self-efficacy than the experimental group. Additionally, participants in the control group experienced the IHT procedure to be easier and reported more positive affect than in the experimental group. There was no significant difference for perceived arousal ratings. Table 3 presents the* t*-tests for the between-group differences following the IHT procedure.

**Table 3:** Means and Independent-Samples t-Test Comparisons for the Five Manipulation Check Items

	Control Group	Experimental Group	*t*	*p*-value
Sense of control	5.95	1.71	21.22	< 0.001
Self-efficacy	6.13	1.79	22.30	< 0.001
Difficulty	1.80	5.85	-20.32	< 0.001
Affective state	5.40	3.62	6.03	< 0.001
Arousal	4.25	3.98	0.83	0.41

### Reaction times in the flanker task

As in Experiment 1, the RT analyses did not include trials with incorrect responses and outliers (3.9%), defined as RT below 200 ms or above 1500 ms (0.9%). As in Experiment 1, RTs for the ten experimental trial blocks were analyzed using a mixed ANOVA with Congruency (congruent vs. incongruent), Proportion Congruent (HPL vs. LPL) and Learning Phase (learning vs. re-learning) as within-participants factors, and the group variable, which included the controllability manipulation (high vs. low control). Again, the main effect of Congruency was significant, *F*(1, 72) = 629.51, *p* < 0.001, *η*^*2*^_*p*_ = 0.907, as well as the interaction Proportion Congruent x Congruency, *F*(1, 72) = 13.03, *p* < 0.001, *η*^*2*^_*p*_ = 0.153, the latter indicating the presence of the CSPC effect. The difference between congruent and incongruent trials was 69 ms in the HPL context and 61 ms in the LPL context, indicating an overall 8 ms CSPC effect (see Table 4). Significant effects were also observed for the variables Group, *F*(1, 72) = 5.69, *p* = *0.0*20, *η*^*2*^_*p*_ = 0.073, and Learning Phase, *F*(1, 72) = 4.84, *p* = 0.031, *η*^*2*^_*p*_ = 0.063, as well as the interaction between these two factors, *F*(1, 72) = 6.79, *p* = 0.011, *η*^*2*^_*p*_ = 0.086. This interaction reflected slower RTs for the experimental than the control group in the Re-Learning phase, *F*(1, 72) = 8.78, *p* = 0.004, *η*^*2*^_*p*_ = 0.109, whereas this slower responding in the experimental group was marginally significant in the Learning phase, *F*(1, 72) = 2.88, *p* = 0.094, *η*^*2*^_*p*_ = 0.038.

**Table 4:** Reaction Times and Error Rates as a Function of Group, Learning Phase, Trial Type, and Proportion Congruency

		Learning Phase						Re-learning Phase						
		HPC		LPC		CSPC		HPC		LPC			CSPC	
		RT	ER	RT	ER	RT	ER	RT	ER	RT	ER	RT		ER
CG	CON	482	0.7	490	1.1			483	0.8	485	0.6			
	INC	556	5.0	543	3.7			550	5.1	549	4.2			
	CE	74	4.3	53	2.6	21	1.7	67	4.3	64	3.6	3		0.7
EG	CON	511	0.8	510	0.2			526	0.8	526	1.2			
	INC	578	4.7	572	4.9			593	7.8	590	4.5			
	CE	67	3.9	62	4.7	5	-0.8	67	7.0	64	3.3	3		3.7

*CG* Control Group, *EG* Experimental Group, *CON* Congruent trial, *INC* incongruent trial, *ACC* Accuracy, *HPC* High proportion congruent, *LPC* Low proportion congruent, CE Congruency Effect, *CSPC* Context-Specific Proportion Congruency effect. Reaction times (RTs) and error rates (ERs) are presented for each group (control vs. experimental) as a function of learning phase (learning vs. re-learning), trial type (congruent vs. incongruent), and proportion congruency location (high vs. low proportion congruent). Congruency effects (CE; congruent − incongruent trials) and context-specific proportion congruency effects (CSPC; CE in high proportion congruent minus CE in low proportion congruent locations) are also reported

**Fig. 2:**
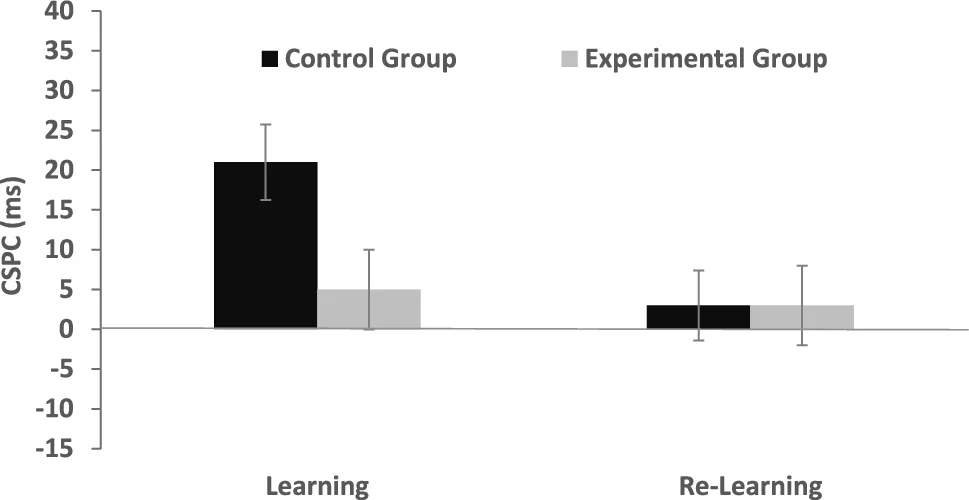
Context-Specific Proportion Congruency (CSPC) Effects as a Function of Control Condition and Learning Phase. Note. Error bars represent standard error of the mean (SEM)

The three-way interaction Group x Proportion Congruent x Congruency was only marginally significant,* F*(1, 72) = 3.12, *p* = 0.082, *η*^*2*^_*p*_ = 0.041. As shown on Fig. 2, this seems to be due to performance in the Learning Phase. In that phase, the CSPC index was 21 vs 5 ms for control and experimental groups respectively, whereas in the Re-Learning Phase was only 3 ms for both groups. The interaction Phase x Proportion Congruent x Congruency fell also close to significance, *F*(1, 72) = 3.76, *p* = 0.056, *η*^*2*^_*p*_ = 0.050, pointing out that the general effect of CSPC found in this experiment might be attributed to the learning obtained in the first phase. In fact, the Proportion Congruent x Congruency interaction was significant in the Learning Phase, *F*(1, 72) = 14.09, *p* < 0.001, *η*^*2*^_*p*_ = 0.164, but it was not present for the Re-Learning Phase, *F*(1, 72) = 1.22, *p* = 0.274, *η*^*2*^_*p*_ = 0.017.

Finally, although the four-way interaction Group x Learning Phase x Proportion Congruent x Congruency was not significant, *F*(1, 72) = 2.77, *p* = 0.100, *η*^*2*^_*p*_ = 0.037, given the marginal effects previously presented and the hypotheses that have driven this study, we compared the CSPS index shown by each group in each learning phase. In the Learning Phase the interaction Group x Proportion Congruent x Congruency was significant, *F*(1, 72) = 5.48, *p* = 0.022, *η*^*2*^_*p*_ = 0.071, which is coherent with our hypothesis of a lower CSPC effect in the experimental group than control group. Specifically, the Control group showed a significant CSPC effect of 21 ms, *F*(1, 39) = 20.12, *p* < 0.001, *η*^*2*^_*p*_ = 0.340, whereas the Experimental group did not reveal the CSPC effect since the index of 5 ms was very small (*F* < 1). Regarding the Re-learning phase, the three-way interaction was not significant (*F* < 1) since, as stated above, in this phase the CSPC index was only 3 ms for each group. All the remaining non-significant effects were* p* > 0.17.

A one-tailed Student’s t-test performed to compare the CSPC index for the Control and Experimental groups in the Learning phase revealed a significant between group difference, *t*(72) = 2.34, *p* = 0.022, *d* = 0.55. Therefore, considering the results of the sensitivity analysis reported above (*d* = 0.59), Cohen’s *d* was close to the minimum effect size that could be detected. In the Re-Learning phase, the difference between the groups was not significant, *t*(72) = - 0.002, *p* = 0.998, *d* = - 0.001.

The same conclusions can be stated by means of Bayesian statistics analyses, showing support for a between groups difference in the Learning Phase, BF10 = 2.433, and evidence in favor of the non-difference hypothesis in the Re-Learning Phase, BF10 = 0.241.

### Accuracy in the flanker task

A mixed ANOVA was conducted for errors rated including the same factors as for RT analyses. This analysis revealed significant effects for Congruency, *F*(1, 72) = 76.60, *p* < 0.001, *η*^*2*^*p* = 0.515, Proportion Congruent, *F*(1, 72) = 6.52, *p* = 0.013, *η*^*2*^_*p*_ = 0.083, and the Congruency x Proportion Congruent interaction, *F*(1,72) = 5.25, *p* = 0.025, *η*^*2*^_*p*_ = 0.068. Thus, the CSPC effect was also present in the accuracy measure. The four-way interaction Phase x Congruency x Proportion Congruent x Group also reached significance, *F*(1, 72) = 5.53, *p* = 0.021, *η*^*2*^_*p*_ = 0.071. However, subsequent ANOVAs conducted on each Phase with the factors Congruency x Proportion Congruent x Group did not show reliable effects, neither in the Learning Phase, *F*(1, 72) = 2.75, *p* = 0.102, *η*^*2*^_*p*_ = 0.037, nor the Re-Learning Phase, *F*(1, 72) = 3.13, *p* = 0.081, *η*^*2*^_*p*_ = 0.042. All the remaining non-significant effects were *p* > 0.14, except the marginally significant effects of Phase, *F* (1, 72) = 3,56, *p* = 0.06, *η*^*2*^_*p*_ = 0.047, and the interaction Group x Phase, *F* (1, 72) = 3,01, *p* = 0.09, *η*^*2*^_*p*_ = 0.040.

A one-tailed Student’s t-test performed to compare the CSPC index for the Control and Experimental groups did not show significant between group differences, neither in the Learning phase, *t*(72) = 1.66, *p* = 0.102, *d* = 0.39, nor in the Re-Learning phase, *t*(72) = - 1.77, *p* = 0.081, *d* = - 0.41.

The same conclusions can be stated by means of Bayesian Statistic analyses, showing no support for a between group difference neither in the Learning Phase, BF10 = 0.778, nor in the Re-Learning Phase, BF10 = 0.913.

### Inverse efficiency in the flanker task

As in Experiment 1, one ANOVA using the Inverse Efficiency Score (Bruyer & Brysbaert 2011; IES = Mean RT / % correct) was performed. This analysis revealed a significant Phase x Congruency x Proportion Congruent x Group four-way interaction, *F*(1, 72) = 6.19, *p* = 0.015, *η*^*2*^_*p*_ = 0.079. Importantly, the ANOVA performed in the Learning phase showed a significant Congruency x Proportion Congruent x Group interaction, *F*(1, 72) = 4.62, *p* = 0.035, *η*^*2*^_*p*_ = 0.093. In this phase, only the control group showed a CSPC effect, *F*(1, 39) = 18.41, *p* < 0.001, *η*^*2*^_*p*_ = 0.321 (*F* < 1 for the experimental group). In the Re-Learning phase, the Congruency x Proportion Congruent was significant, *F*(1, 72) = 8.78, *p* = 0.004, *η*^*2*^_*p*_ = 0.109, but not modulated by Group, *F*(1, 72) = 2.75, *p* = 0.102, *η*^*2*^_*p*_ = 0.037.

A one-tailed Student’s t-test performed to compare the CSPC index for the Control and Experimental groups in the Learning phase revealed a significant between group difference, *t*(72) = 2.15, *p* = 0.035, *d* = 0.50, but not for the Re-Learning phase, *t*(72) = - 1.66, *p* = 0.102, *d* = - 0.39.

Similar conclusions can be drawn by means of Bayesian analyses on the between-group differences in the CSPC effect for IES in each phase. Whereas in the Learning phase evidence supported the fact that the experimental group showed a reduced CSPC effect compared to the control group for, BF10 = 1.708, in the Re-Learning phase evidence supported the lack of between-group differences, BF10 = 0.780.

## Discussion

We observed a lack of the CSPC effect during the learning phase in the experimental group exposed to high uncontrollability. This effect remained absent in the re-learning phase; however, this outcome can be attributed to the fact that, if no association was formed initially, there was nothing to re-learn. Thus, the impact of uncontrollability was primarily confined to the initial learning phase but nonetheless affected overall task performance, indicating a reduced reliance on contextual cues compared to participants whose sense of personal control was enhanced. Interestingly, while both induced uncontrollability, in Experiment 2, and trait anxiety in Experiment 1 influenced the CSPC effect, they did so at different stages: the re-learning phase for participants with high trait anxiety, and both the learning and re-learning phases for those who experienced uncontrollability. These differences and their potential implications are discussed in greater detail in the General Discussion.

It is important to note, however, that a decrease in the CSPC effect was also observed in the control group, which was an unexpected finding. This pattern may be attributable to mental fatigue and reduced attentional focus induced by completing the IHT procedure, which involves cognitively demanding reasoning and inferential processes even in the control group, as well as by performing the learning phase of the CSPC task. However, this interpretation remains post hoc, and it is also possible that the observed effect reflects an artefact specific to the re-learning phase of the task. If so, the results from the re-learning phase should be interpreted with caution.

Additionally, it should be acknowledged that the main CSPC effect was smaller than the minimum detectable effect size estimated in the sensitivity analysis and should therefore be interpreted cautiously. Future replications of this study should focus specifically on the learning phase in order to test whether uncontrollability selectively affects context-dependent learning in the allocation of attentional control.

## General discussion

Constantly changing and volatile environments can threaten basic needs for certainty, predictability and control (Bukowski and Kofta 2017; Jonas et al. 2014). When environmental contingencies between actions and outcomes are unclear, individuals may develop an anxious response, characterized by over-vigilance and a more stimulus-driven mode of attentional control (Corbetta & Shulman; 2002). Prolonged exposure to unpredictability and uncontrollability can further lead to a withdrawal of active coping responses, resulting in a more dispersed attentional focus and impairments in goal-driven attentional control and cognitive flexibility (Bukowski & Kofta 2017; Bukowski et al. 2024). In the present research, we hypothesized that similar underlying cognitive mechanisms – specifically, flexible incidental learning from environmental cues – might be disrupted in both chronically highly anxious individuals and those exposed to prolonged episodes of uncontrollability. More specifically, we tested whether these groups exhibit impaired learning from contextual cues that signal when attentional control should or should not be allocated. Overall, our findings indicate that both conditions negatively affect the contextual allocation of cognitive control.

Interestingly, and contrary to our initial predictions, we found distinct patterns of results for the two groups. Specifically, the high-anxiety group did not exhibit difficulties in incidental learning during the initial phases of the procedure, demonstrating a CSPC effect that reflects the ability to learn from task context. However, when the context changed, they showed a decrement in flexible learning, as indicated by a decline in the CSPC effect during the re-learning phase. In contrast, individuals exposed to uncontrollability (action–outcome non-contingency learning) displayed impairments in incidental learning from the very first phase of the task.

These findings suggest that different mechanisms underlie performance in the high-anxiety and high-uncontrollability groups. In anxious participants, we observed heightened sensitivity to cues signaling how to perform the task more efficiently (i.e., when and where to apply greater or lesser attentional control). Conversely, control-deprived participants appeared insensitive to implicit contextual cues that could facilitate learning. These results are consistent with prior research indicating that trait anxiety is associated with increased responsiveness to environmental signals (stimulus-driven control). Such sensitivity can be advantageous in certain instances of incidental learning - when there is no explicit intention to learn - but may also lead to greater distractibility and reduced flexibility, which are necessary for acquiring new associations that require more effortful processing (Eysenck et al. 2023).

Prolonged exposure to uncontrollability, however, resulted in a reduced tendency to learn from contextual cues from the outset of the Flanker task. This finding suggests that previous unsuccessful learning experiences – specifically, related to the absence of action–outcome contingencies – may impair the readiness to learn from the task context. This impairment could result from a shift from externally focused attention to internally focused attention, associated with off-task cognitions and ruminative thoughts (Mikulincer 1989). These results imply a disconnection from the environment: when personal control is lacking, attention may become concentrated on internal processes, while external processing becomes more disorganized, reflecting a dysregulated balance between internally and externally directed attention (Bukowski et al. 2024).

The findings further advance our understanding of the effects of uncontrollability on basic cognition, revealing after-effects on incidental, and, to some extent, implicit learning. A lack of personal control appears to hinder flexible learning processes that do not rely on voluntary or intentional processing. Similarly, recent evidence indicates that induced and prolonged uncontrollability can disrupt the implicit sense of agency (Soral et al. 2021). Consequently, the after-effects of uncontrollability may extend beyond the scope of conscious processing. This research demonstrates that motivational states, such as the aversive experience of lacking personal control, can influence non-intentional forms of learning related to the allocation of cognitive control. Overall, these findings suggest that previously observed deficits in attentional control may arise from a reduced reliance on contextual cues, which in turn impairs flexible learning.

Alternatively, a motivational explanation also appears plausible, suggesting that experiences of control deprivation can reduce individuals’ engagement in the task at hand. Reaction time data from Experiment 2 indicated that, following prolonged control deprivation, participants responded more slowly overall, which may reflect reduced vigilance and attentional focus. Impaired learning during the first phase of the task further supports the interpretation that participants exposed to uncontrollability paid less attention to contextual cues. Prior research has shown that lack of control can hinder flexible learning of new contingencies and categorization rules (Bukowski et al. 2024). In a task environment where contextual cues can guide the application of attentional control, individuals deprived of control may fail to learn the association between context and the Flanker task from the outset. Thus, both motivational factors (e.g., reduced task involvement) and cognitive mechanisms (e.g., unbalanced action-control strategies, such as exploitation versus exploration) may have jointly contributed to the observed pattern of results.

A novel contribution of this research lies in its focus on incidental and potentially implicit learning mechanisms in individuals experiencing anxiety and uncontrollability. Implicit learning from the environment relies on automaticity, allowing environmental cues to guide behavior. In this context, incidental learning does not depend on effortful control; rather, it requires selective attention to predictive cues, even when such attention is not explicitly directed toward learning (Jiménez & Méndez, 1999). For example, the location of a target is relevant for task performance and simultaneously serves as a cue for learning about contingencies. Learning the relationship between location (upper or lower) and proportion congruency can occur because participants selectively attend to the location to complete the task, without requiring effortful processing of the contingency itself. Thus, implicit learning emerges as an automatic by-product of how selective attention is allocated.

Our research demonstrates that dispositional anxiety and prolonged exposure to lack of control can impair not only effortful attentional control but also more automatic learning processes that rely on contextual cues, which support the efficient allocation of cognitive control. In this context, hyper-vigilance in highly anxious participants may not have initially hindered learning about contextual cues; rather, it appears to have increased rigidity, reducing sensitivity to changes in those cues. In contrast, induced uncontrollability disrupted selective attention and associative processes from the outset, preventing implicit learning of predictive cues. These findings also extend the literature on the influence of affective and motivational states on implicit learning (Eitam et al. 2009; Popławska-Boruc et al. 2019), demonstrating that aversive experiences can reduce sensitivity to contextual cues that facilitate flexible learning.

Finally, the effects of induced uncontrollability can be understood dynamically. Initial reactions to uncontrollability often involve increased information seeking and environmental exploration (Mikulincer 1994). While this strategy may facilitate the discovery of new solutions, it can also increase distractibility and overload, producing behavioral uncertainty and difficulties in selecting effective action strategies (Bukowski & Kofta 2017). An initial bias toward exploration over exploitation may thus characterize responses to changing and volatile environments. However, prolonged exposure to uncontrollability can promote a more rigid processing mode, emphasizing the exploitation of existing strategies and persistence at the expense of flexibility (Bukowski et al. 2024; Hommel 2015). These two processing modes appear to underlie the differential outcomes observed in anxious individuals versus those experiencing a lack of personal control, particularly in flexible learning about when to allocate attentional control. Future research should investigate how uncontrollability affects implicit learning and adaptive cognitive control, with particular attention to the dynamics and temporal progression of coping responses, from anxious vigilance to rigid, ruminative processing.

This research has several limitations, primarily they consist in the largely exploratory nature of the studies, lacking a proper preregistration protocol and a-posteriori statistical power analyses. Sensitivity analyses revealed that our samples are not sufficiently well-powered, especially for the conducted interaction analyses. Nevertheless, these initial findings provide valuable insights into the effects of anxiety and uncontrollability on incidental learning processes, paving the way for future studies to replicate and extend the results. Subsequent research should clarify the conditions under which anxiety and uncontrollability produce divergent after-effects on context-dependent allocation of cognitive control.

In addition, it would be worthwhile in future research to consider the distinction between trait and state anxiety, and to treat trait anxiety as a continuous variable rather than dividing the sample into high- and low-anxiety groups. Moreover, the finding that effects of the control manipulation emerged only during the learning phase may be attributable to the typically strong but relatively short-lived nature of induced uncontrollability effects, with carry-over effects often limited to early phases of subsequent tasks (Bukowski et al. 2026). Accordingly, future studies should examine the role of perceived lack of control both as a situationally induced state and as a dispositional characteristic (e.g., individual differences in perceived control or self-efficacy).

Beyond these limitations, the present study offers novel insights into how chronic anxiety and prolonged exposure to uncontrollability may influence fundamental cognitive control mechanisms. In particular, it raises new questions for future research on the context-dependent learning of attentional control allocation. By revealing distinct patterns across these two conditions, our findings also contribute to refining current theoretical models of cognitive flexibility in the context of anxiety and uncontrollability. These contributions not only advance the understanding of adaptive and maladaptive attentional processes but may also inform the development of targeted interventions to enhance attentional regulation in clinical and educational contexts.

## Data Availability

The data is available on OSF and linked to the following address: https://osf.io/7jv26/overview?view_only=5ff509b75c1c46a7ba47c6f71d5be48e.

## Footnotes

1 We have also performed a sensitivity analysis for the 3-way interaction, Group x Proportion Congruent x Congruency, which showed that the minimum effect size that could be detected for α = 0.05 and β = 0.80 is Cohen’s *f(V)* = .455. This effect size corresponds to a *η*^*2*^_*p*_ = .17.

2 We have also performed a sensitivity analysis for the three-way interaction, Group x Proportion Congruent x Congruency, which showed that the minimum effect size that could be detected for α = 0.05 and β = 0.80 is Cohen’s *f(V)* = .387. This effect size corresponds to a *η*^*2*^_*p*_ = .13.
